# Physicochemical study of natural fractionated biocolloid by asymmetric flow field-flow fractionation in tandem with various complementary techniques using biologically synthesized silver nanocomposites

**DOI:** 10.1007/s00216-018-0967-0

**Published:** 2018-04-03

**Authors:** Viorica Railean-Plugaru, Pawel Pomastowski, Tomasz Kowalkowski, Myroslav Sprynskyy, Boguslaw Buszewski

**Affiliations:** 1 Department of Environmental Chemistry and Bioanalytics, Faculty of Chemistry, Nicolaus Copernicus University, Gagarina 7, 87-100 Torun, Poland; 2 Centre for Modern Interdisciplinary Technologies Nicolaus Copernicus University, Wileńska 4, 87-100 Torun, Poland

**Keywords:** Biologically synthesized silver composites, Asymmetric flow field-flow fractionation, Fractionation, Organic deposit, Matrix-assisted laser desorption ionization mass spectrometry, Silver clusters

## Abstract

Asymmetric flow field-flow fractionation coupled with use of ultraviolet–visible, multiangle light scattering (MALLS), and dynamic light scattering (DLS) detectors was used for separation and characterization of biologically synthesized silver composites in two liquid compositions. Moreover, to supplement the DLS/MALLS information, various complementary techniques such as transmission electron spectroscopy, Fourier transform infrared spectroscopy, and matrix-assisted laser desorption ionization time-of-flight mass spectrometry (MALDI-TOF MS) were used. The hydrodynamic diameter and the radius of gyration of silver composites were slightly larger than the sizes obtained by transmission electron microscopy (TEM). Moreover, the TEM results revealed the presence of silver clusters and even several morphologies, including multitwinned. Additionally, MALDI-TOF MS examination showed that the particles have an uncommon cluster structure. It can be described as being composed of two or more silver clusters. The organic surface of the nanoparticles can modify their dispersion. We demonstrated that the variation of the silver surface coating directly influenced the migration rate of biologically synthesized silver composites. Moreover, this study proves that the fractionation mechanism of silver biocolloids relies not only on the particle size but also on the type and mass of the surface coatings. Because silver nanoparticles typically have size-dependent cytotoxicity, this behavior is particularly relevant for biomedical applications.

**Electronic supplementary material:**

The online version of this article (10.1007/s00216-018-0967-0) contains supplementary material, which is available to authorized users.

**Graphical abstract:**
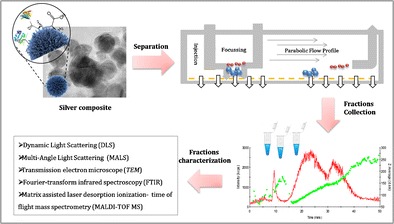
Workflow for asymmetric flow field-flow fractionation of natural biologically synthesized silver nanocomposites

## Introduction

The development of biologically inspired experimental processes for the synthesis of silver nanoparticles (AgNPs) has grown into a powerful branch of nanotechnology. Applications in the field of medicine include the formulation of many potential antimicrobial agents that are effective against many human pathogens, including multidrug-resistant bacteria. Multidrug-resistant strains of bacteria have become a serious problem of public health. In this field, novel approaches are required to develop new therapeutic agents or to modify available ones to combat resistant pathogens.

The silver ions released as a secondary oxidation product of AgNPs are toxic to the cells of microorganisms. Because of this, great attention has been focused on the size and concentration toxicity of standard AgNPs. The size of AgNPs influences their physicochemical properties, and thus mechanisms of their toxic action, and in consequence their bioavailability. Good distribution of small nanoparticles (10 nm) after intravenous administration of gold nanoparticles (10, 50, 100, and 250 nm) has been reported in almost all organ systems (e.g., blood, kidney, lung, testis) [1]. Many results have shown that toxicity increases with decreasing particle size but some of them established the opposite or no dependence of toxicity with size [2, 3]. Many studies discussed the importance of the organic matrix on the surface of nanoparticles in influencing toxicity [4, 5]. The impact of a specific number of DNAs on the electrophoretic separation of chemically coated nanoparticles was also reported. In addition to this method, a wide range of analytical techniques were used to provide information on particle size distributions; namely, chromatography [6], laser scattering [7], spectroscopy [8], and field-flow fractionation (FFF) [9]. In comparison with the other methods, FFF is a rapidly emerging technique with increased resolution, sensitivity, and selectivity. It is designed to separate a wide range of analytes, including nanoparticles, colloids, and macromolecules, on the basis of the diffusion mechanism. The average size and size distribution of analytes can differ significantly from one technique to another [10]. For this reason, many studies use FFF systems for separation of standard AgNPs synthesized by a chemical method. Moreover, FFF systems are coupled with a variety of detectors, including inductively coupled plasma mass spectrometry (MS), UV–visible, multiangle laser light scattering (MALLS), and dynamic light scattering (DLS) detectors in online or offline mode [10, 11].

Therefore, in this work we used asymmetric flow FFF (A4F) for fractionation of silver nanocomposites synthesized by a biological method. Moreover, to provide complementary information about the heterogeneous nanoparticles, the A4F system was coupled with UV, MALLS, and DLS detectors. Two types of mobile phase with different ionic strength were used; namely, (1) deionized water and (2) phosphate-buffered saline containing 0.09% sodium azide (pH 7.2). The size distribution and shapes of the particles were determined by transmission electron microscopy (TEM). Moreover, to illustrate the presence of organics, silver isotopes, silver–organic combinations, and clusters of silver in separated fractions, matrix-assisted laser desorption ionization (MALDI) time-of-flight (TOF) MS and Fourier-transform IR (FTIR) spectroscopy were performed. Furthermore, no study has yet investigated biologically synthesized nanoparticles, and the origin of surface organic deposits, using an A4F system. In this regard, since no applicable information is available, it would be highly relevant to be able to perform deep characterization of specific heterogeneous nanomaterials.

## Materials and methods

The biologically synthesized silver nanocomposites used in this research were previously synthesized and characterized by Railean-Plugaru et al. [12].

An AF2000 system for 4AF was purchased from Postnova Analytics. Acquisition of signals was done online with UV–visible (PN3211, Postnova Analytics), MALLS (PN3621, Postnova Analytics), and DLS (Zetasizer Nano ZS, Malvern Instruments) detectors. The wavelength of the UV detector was set at 420 nm to monitor the signal of silver composites. The *Z*-average size (hydrodynamic diameter) of fractionated silver composite fractions was measured in triplicate with use of a quartz flow cell. The AF2000 control software (Postnova Analytics) was used to operate the system, acquire signals from the detectors, and process data from light scattering. The channel was equipped with a 350-μm spacer and a 10-kDa cutoff (Postnova Analytics) regenerated cellulose membrane. Different carrier liquids (water; KCl and 0.87% NaCl; phosphate-buffered saline; phosphate-buffered saline containing 0.09% sodium azide, pH 7.2) were tested to ensure the stability of the AgNPs by measurement of the zeta potential and screening of the changes of their size with use of the Zetasizer Nano ZS system. Deionized water and phosphate-buffered saline containing 0.09% sodium azide (pH 7.2) were finally used for dissolution and fractionation of the sample as the mobile phase because of their lowest and highest recorded stability. The detector flow rate, injection time, cross flow rate, and elution time were also optimized to obtain the maximum number of fractions. The optimized parameters were set for the focusing step (injection flow rate 0.20 mL min^-1^, cross flow rate 0.39 mL min^-1^, injection time 7 min, injection volume 150 μL) and for the elution step (cross flow rate 0.39 mL min^-1^ exponentially decreasing to 0.1 mL min^-1^, elution time 20 min, see Fig. S1). During the fractionation of biologically synthesized silver composites, the detector flow rate was constant (0.5 mL min^-1^). The concentration of each injection was 106.8 μg mL^-1^. Fractions were collected manually in Eppendorf vials and then analyzed by FTIR spectroscopy, TEM, and MALDI-TOF MS.

### FTIR analysis

The experiments were conducted by addition of 2 μL of sample solution per membrane position for each Direct Detect® assay-free sample card (catalog no. DDAC00010-81) with use of the thin layer method in a Direct Detect® IR spectrometer (Merck Millipore, Germany). The Direct Detect® spectrometer collected the mid-IR spectrum of the energy that passes through the protein sample. The FTIR spectra were recorded in the range from 1350 to 1870 cm^-1^.

### Transmission electron microscopy

TEM analysis was used to prove the presence of metallic silver and measure the size of particles dispersed in collected fractions. Images for all collected fractions were recorded with an FEI Tecnai F20 X-Twin microscope. The concentrated samples were prepared for imaging by drying of the nanoparticles dropped on a carbon-coated copper grid (Lacey carbon support film 400 mesh, Electron Microscopy Sciences).

### MALDI-TOF/TOF MS analysis

The matrix [α cyano-4-hydroxycinnamic acid (HCCA)] and silver samples were deposited on ground steel targets (Bruker Daltonics, Bremen, Germany) by the spotted dried droplet method [13]. All reagents were purchased from Fluka Feinchemikalien (Neu-Ulm, Germany). The experiments were performed with a MALDI-TOF/TOF mass spectrometer (Bruker Daltonics, Bremen, Germany) in tandem with a modified neodymium-doped yttrium aluminium garnet laser operated at 355 nm and 2 kHz.

Protein Calibration Standards I (Bruker Daltonics, Bremen, Germany) and HCCA were used for calibration:

## Results

### Size distribution of biologically synthesized silver composites

**Fig. 1:**
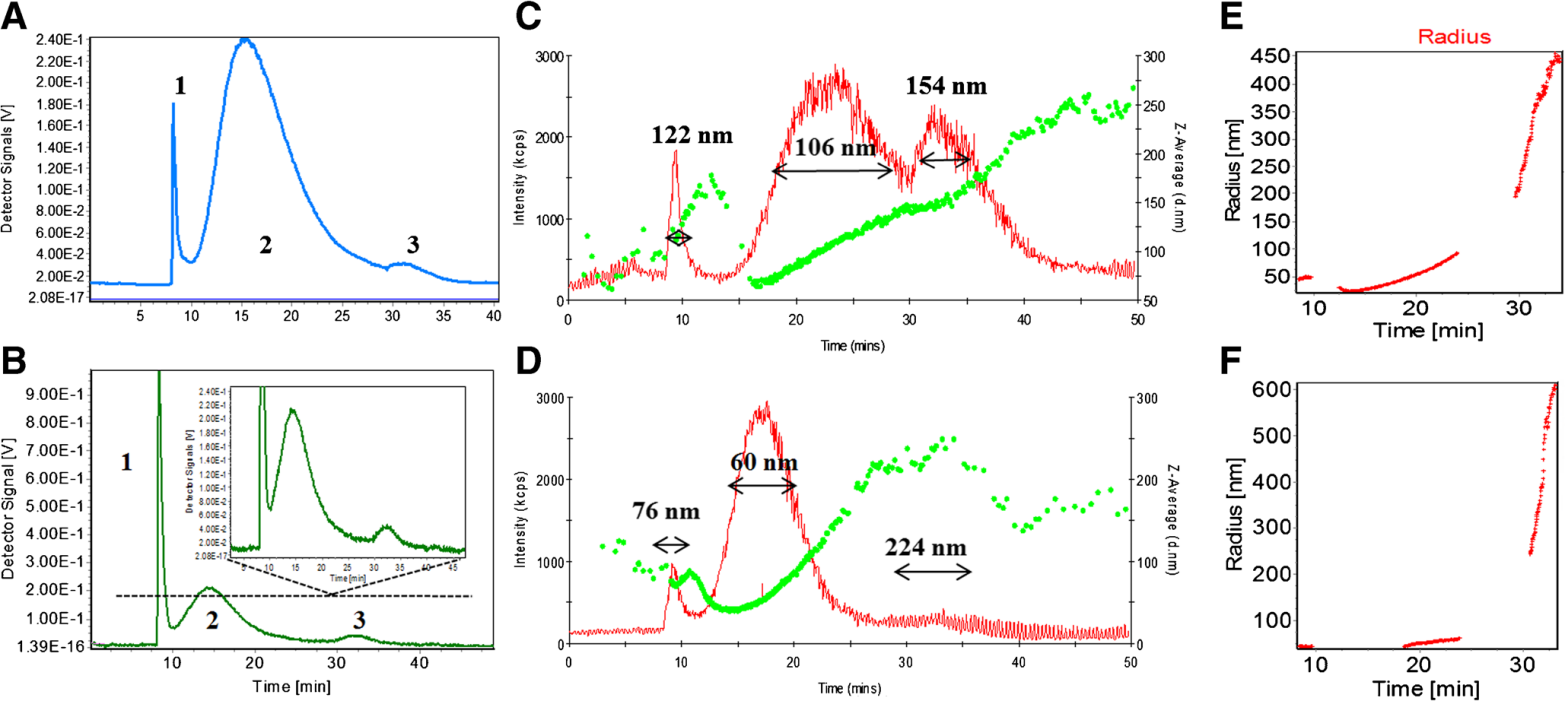
Size distribution of biologically synthesized silver nanoparticles in water (a) and phosphate-buffered saline containing 0.09% sodium azide (b), hydrodynamic diameter of the particles measured by dynamic light scattering in water (c) and in phosphate-buffered saline containing 0.09% sodium azide (d), and radius of gyration of silver composites measured by multiangle laser light scattering in water (e) and in phosphate-buffered saline containing 0.09% sodium azide (f)

The fractograms shown in Fig. 1a and b illustrate the effect of the carrier liquid composition on the size distribution of the silver biocolloids. The concentration signal obtained with the UV–visible detector varied in intensity in the case of water and 0.09% sodium azide in phosphate-buffered saline. The hydrodynamic effective radius of the particles measured by DLS is shown in Fig. 1c and d. The size of the separated silver composites indicated a mixed mode of elution. The mean values of the particle diameter were 122 nm (1), 106 nm (2), and 154 nm (3) in the case of water (Fig. 1c) and 76 nm (1), 60 nm (2), and 224 nm (3) in the case of 0.09% sodium azide in phosphate-buffered saline (Fig. 1d). Additionally, the radius of gyration of the silver composites measured by MALLS was 90–100 nm (1), 20–120 nm (2), and 200–500 nm (3) in the case of water (Fig. 1e) and 45–50 nm (1), 45–60 nm (2), and 250–600 nm (3) in the case of 0.09% sodium azide in phosphate-buffered saline (Fig. 1f).

**Fig. 2:**
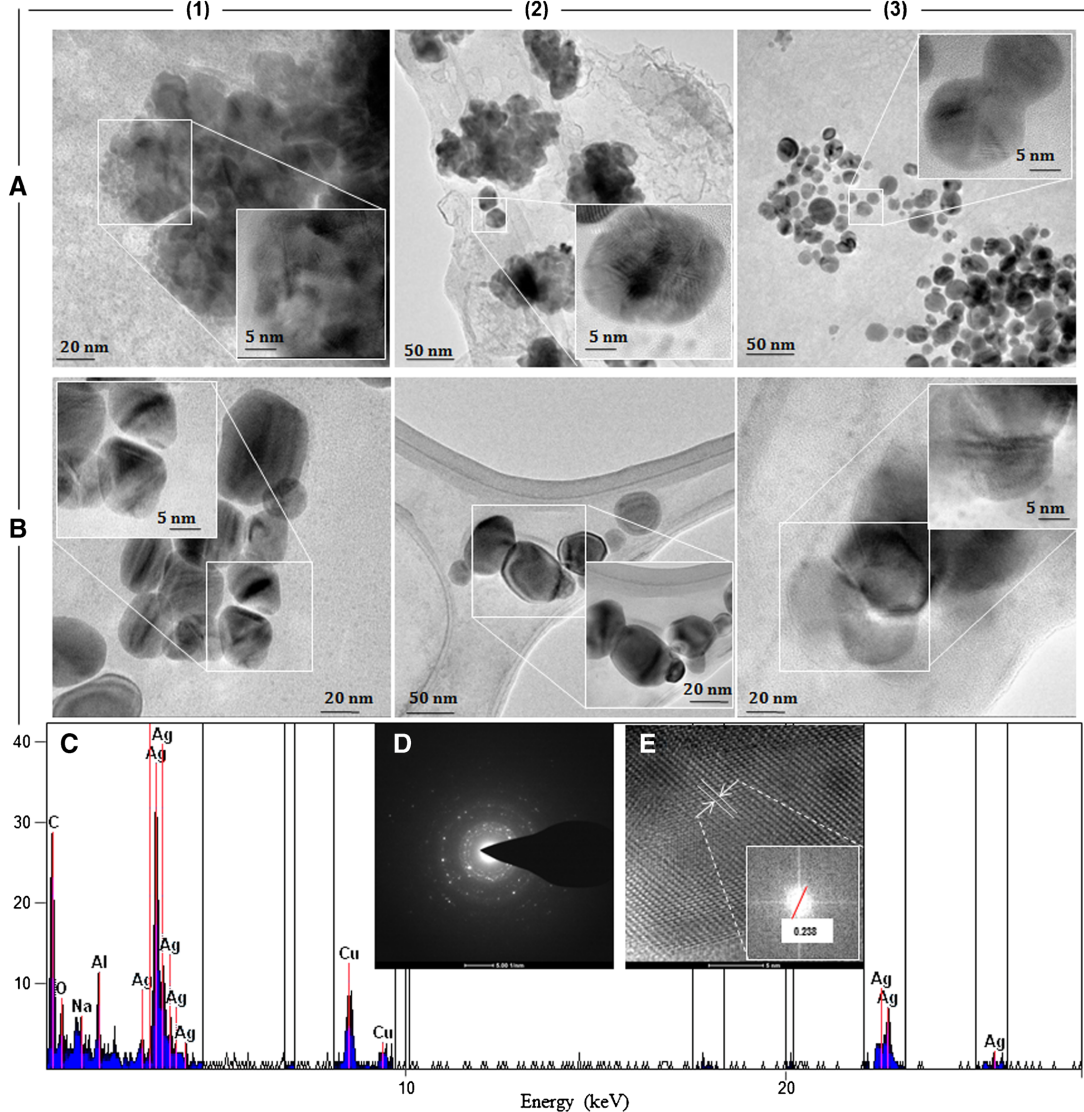
Transmission electron microscopy (TEM) images of biologically synthesized silver nanoparticles in separated fractions in water (A) and in phosphate-buffered saline containing 0.09% sodium azide (B), energy-dispersive X-ray spectrum (C), selected-area electron diffraction image (D), and high-resolution TEM image (E)

The collected fractions were subjected to TEM analysis. TEM can not only yield information about the size of individual particles directly observed but also provides the distribution and morphology of nanoparticles to judge whether agglomerates are present in the sample. Figure 2 illustrates TEM images of AgNP separated fractions in water (panel A) and 0.09% sodium azide (panel B). The TEM images look strikingly different from each other. The images show typical agglomeration for both fraction 1 (panel A) and fraction 2 (panel A) and good dispersion of AgNPs for fraction 3 (panel A) in the case of water. By manual image analysis, the diameter of silver dispersed particles (fraction 3, Fig. 2, panel A) was estimated to be 4–45 nm (Fig. 2, panel A). In contrast, the TEM images of collected fractions in 0.09% sodium azide (Fig. 2, panel B) do not show large agglomeration for these samples. In this case the AgNPs are polydisperse with roughly spherical shape and with size ranging between 10 and 30 nm for fractions 1and 2, whereas fraction 3 contains clusters of about 50 nm. Strong signals of silver of approximately 3 keV in the energy-dispersive X-ray spectrum confirmed the presence of elemental silver (Fig. 2, panel C). The minor amounts of carbon and oxygen can be assigned to the X-ray emission from proteins and carbohydrates attached to the silver core of silver composites [14]. The presence of copper signals comes from the TEM grid. Selected-area electron diffraction (Fig. 2, panel D) confirmed the highly crystalline nature of the synthesized nanoparticles in each collected fraction. In both solutions and for all of the collected fractions, high-resolution TEM showed interference fringe patterns with interplanar distances of 0.238 nm, which correspond to the silver distance of atomic layers (Fig. 2, panel E).

### FTIR analysis

**Fig. 3:**
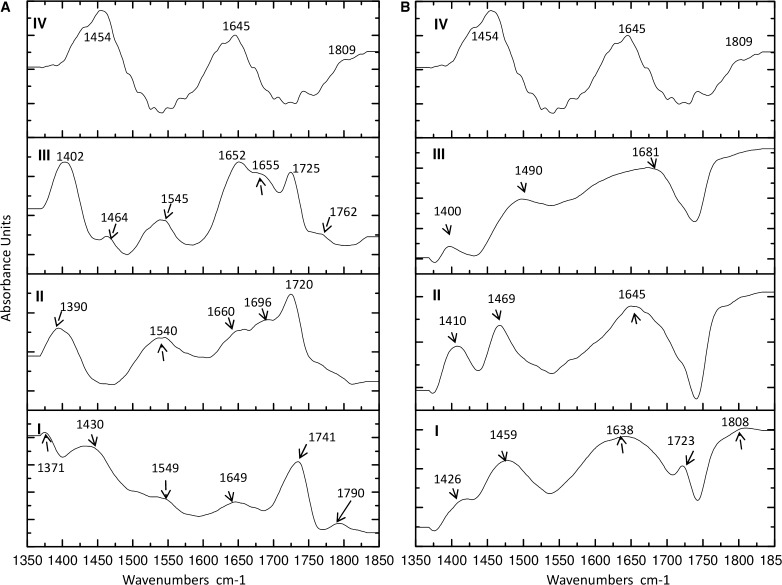
Fourier transform IR spectra of collected fractions after separation (I, II, III) and biologically synthesized silver nanocomposites before separation (IV) in water (**a**) and in phosphate-buffered saline containing 0.09% sodium azide (**b**)

Taking into account the vibrational changes seen in the FTIR spectra at different pH values in our previous work [12], we used spectroscopy to identify the functional groups localized into/onto organic deposits of the silver biocolloids in samples before separation and to monitor the changes of the branch size of surface organic deposits of silver nanocomposites after fractionation. The FTIR spectra of the collected fractions after fractionation and of AgNPs before separation in the case of water and 0.09% sodium azide in phosphate-buffered saline are presented in Fig. 3. The spectra illustrate the distribution of organic deposits and provide a base for comparison of differences between analyzed fractions. In the case of water, the signals obtained for all fractions contain absorption bands at 1720 and 1725 cm^-1^ resulting from C=O vibration, which could correspond to aspartate and glutamate [15], and an absorption band at 1741 cm^-1^ (Fig. 3a, fraction I), corresponding to the –COO^-^ stretching vibration [16]. The absorption peak localized at 1649 cm^-1^ (Fig. 3a, fraction I) is related to C=O vibrations in asparagine, and the peaks at 1652 and 1660 cm^-1^ are related to C=O vibrations in arginine [16]. Moreover, the same position of the absorbance maximum confirms the presence of α-helices (1649 and 1652 cm^-1^) and 3_10_-helices (1660 cm^-1^) in proteins and polypeptides [17]. The bands at 1540, 1545 cm^-1^ and 1549 cm^-1^ (Fig. 3a, I–III) correspond to bending vibration of amide II, specially the vibration of β-sheet and δN–H, νC–N bonds [18]. The absorption bands at 1371 and 1390 cm^-1^ (Fig. 3a, fractions I and II) originate from coupling of adjacent CH_2_ groups with tyrosine OH, and the band at 1402 cm^-1^ originates from symmetric stretching vibration of the –COO^-^ group of aspartate [13].

New signals at 1762 and 1790 cm^-1^ from protonated carboxyl groups appeared only in fractions III and I, respectively. Additionally, new bands near 1655 cm^-1^ (fraction III) and 1696 cm^-1^ (fraction II) (Fig. 3a) were assigned to antisymmetric stretching vibration of CN_3_H_5_^+^ originating from arginine [17]. Another new band, originating from CN stretching vibration of tryptophan, was also reported by earlier workers at 1464 cm^-1^ (Fig. 3a, fraction III) [19].

In the case of 0.09% sodium azide, the absorption band at 1426 cm^-1^ (Fig. 3b, fraction I) is a consequence of the presence of amide II vibrations originating mainly from the NH bending and CN stretching vibrations in the HisH structure, whereas COO^-^ symmetric stretching and CN stretching from the acidic amino acids aspartate, glutamate, and glutamine occurred at 1400 and 1410 cm^-1^, respectively (Fig. 3b, fractions II and III) [15, 18]. Bands at 1459 and 1469 cm^-1^ (Fig. 3b, fractions I and II) are assigned to CN vibration of proline. The band at 1490 cm^-1^ (Fig. 3b, fraction III) is assigned to CC ring stretching in phenylalanine [15]. The side chain absorbance in the range from 1638 to 1681 cm^-1^ is related to the amide I group, and is caused by stretching of β-sheet structure (1638 cm^-1^), C=C in uracil, NH_2_ in guanine (1645 cm^-1^), and C=O vibration that are hydrogen bonded (1681 cm^-1^) [18]. A new absorption band (1723 cm^-1^) appeared only in fraction I (Fig. 3b), and is attributed to the formation of dimers through hydrogen bonds that weaken the carbonyl bond [20].

### MALDI-TOF/TOF MS analysis

In our previous research [12] MALDI-TOF/TOF-MS as a complementary technique was used to prove the presence of active functional groups of amino acids in organic deposits and record the silver clusters. In the current study we used the same method for deep characterization of separated fractions and to compare them with native samples to monitor the changes caused by different buffers regarding the branching of surface organic deposits connected with metals for each fraction.

**Fig. 4:**
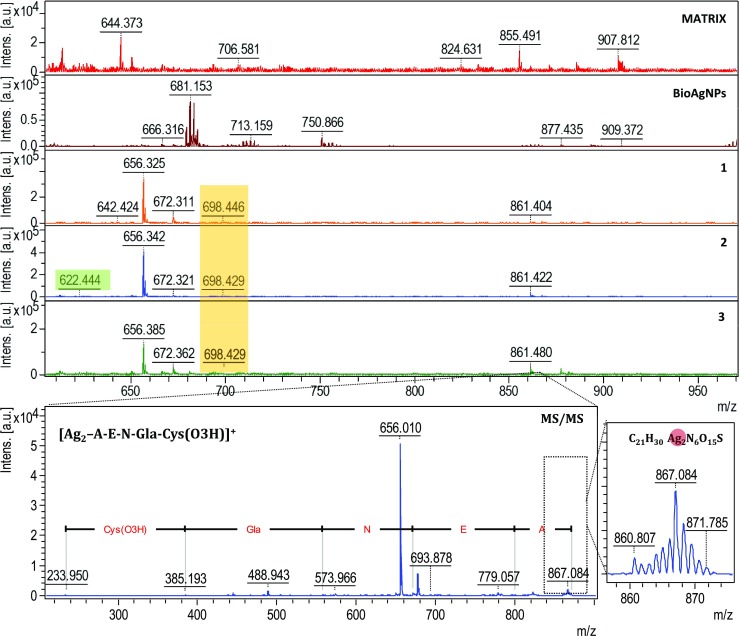
Mass spectra obtained by one- and two-dimensional matrix-assisted laser desorption ionization time-of-flight mass spectrometry (MS), and isotopic distribution zoom of the *m*/*z* = 867 metal–organic signal. BioAgNPs biologically synthesized silver nanoparticles

**Fig. 5:**
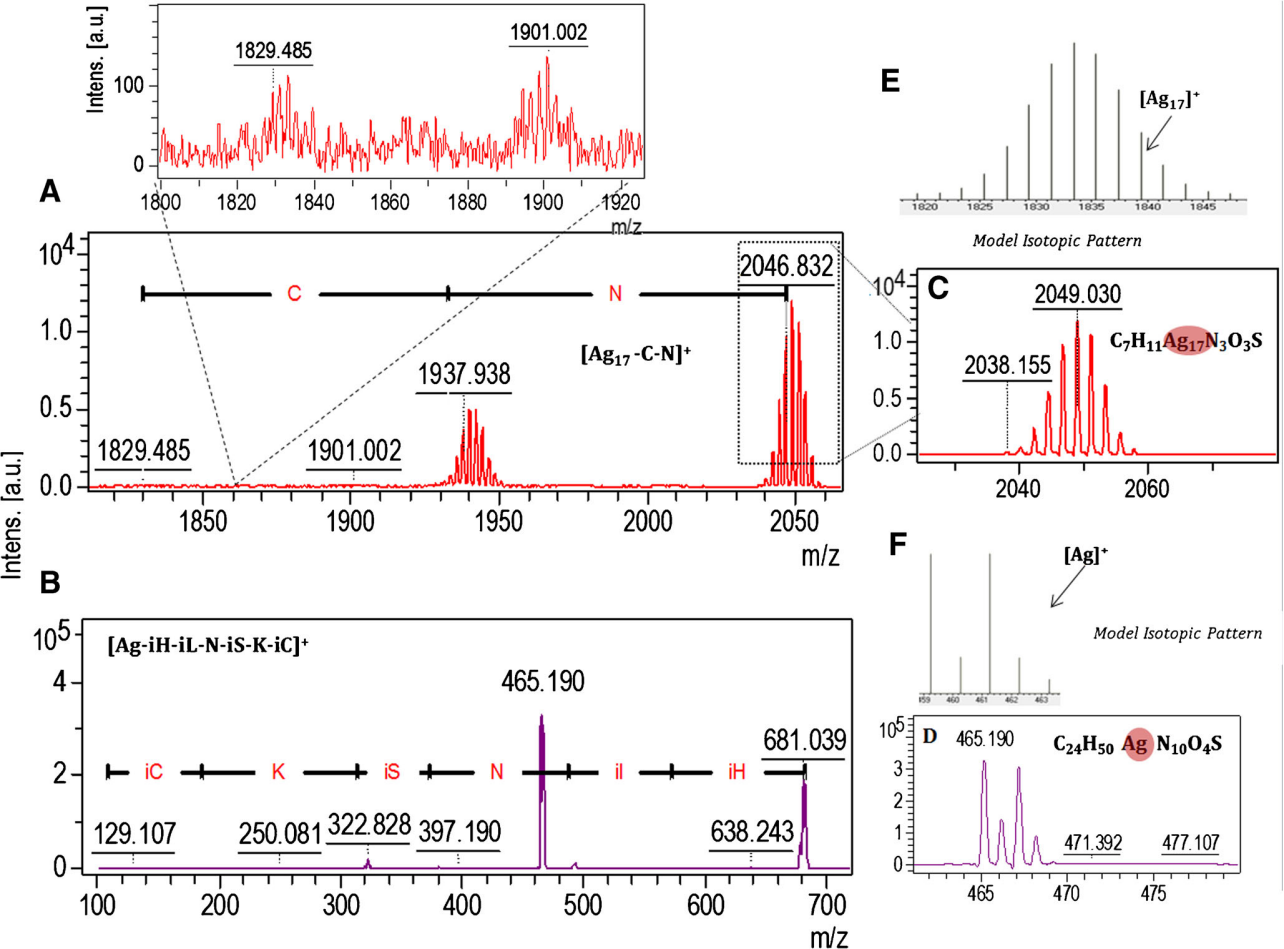
Comparative LIFT spectra of a multi-silver cluster (A) and a one-silver ion (B) combined with organic species, isotopic distribution zoom of selected *m*/*z* = 2046 (C) and *m*/*z* = 465 (D) metal–organic signals of parent ions, and isotopic pattern model of [Ag_17_]^+^ (E)and [Ag]^+^ (F) ions

One-dimensional and two-dimensional MALDI-TOF/TOF MS were performed to identify and compare the presence of silver and its forms in native biological silver mixtures and collected fractions of biologically synthesized silver composites. Figure 4 shows the one-dimensional spectra of HCCA matrix as a control, unfractionated biologically synthesized AgNPs, and three fractions of AgNPs. Comparative analysis of the one-dimensional MALDI MS spectra allowed the signals coming from the unfractionated and fractionated AgNPs to be distinguished (Fig. 4). The characteristic isotopic pattern of silver and use of the LIFT fragmentation approach [21] allowed the identification of metal–organic binding (Fig. 5). Table 1 summarizes the identified signals of two-silver and multi-silver complexes with peptides and amino acids. Silver–organic signal convergence of native and fractionated biologically synthesized AgNPs was observed. Signals at *m*/*z* = 672 and *m*/*z* = 861 present in the native mixture and all AgNP fractions (Table 1) suggest the dominant presence of V-AP-I/LP and IA-E-V-NP-IA peptide sequences in the biological silver samples. On the other hand, signals that are not present in the native mixture of silver were registered in the collected fractions. For instance, the signal at *m*/*z* = 656 was registered only for silver fractions, whereas *m*/*z* = 1078 was observed only for all of the silver fractions collected in phosphate-buffered saline containing 0.09% sodium azide, and the presence of *m*/*z* = 1448, representing Ag_8_-W-HPP was found only in the third silver fraction in the water system (Table 1). The presence of new signals in silver fractions but absence in the mixture of biologically synthesized AgNPs is a result of suppression of the most abundant compounds in the native unfractionated AgNP mixture. As evidence of this, individual and characteristic signals were recorded only for the fractions of AgNPs such as *m*/*z* = 600, 1400, 1118, 1133, 1239, and 1448. Moreover, in the case of the water system, 17% more signals were recorded in comparison with the azide system. The results suggest that various carrier liquids (buffer system) regulate the size of AgNPs and the composition of the organic coating, and thus determine different A4F mechanisms and directly influence separation.

**Table 1:**
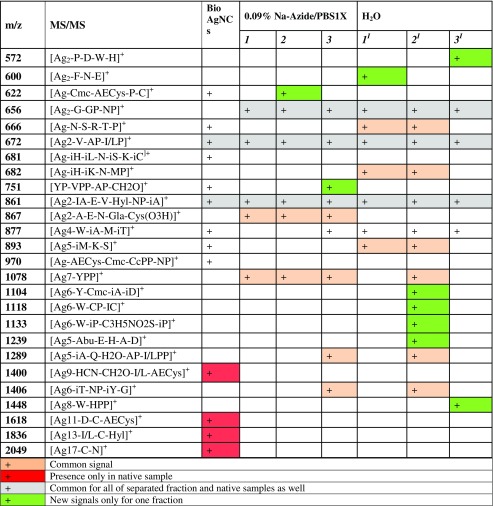
Characteristic signals identified for the native sample (biologically synthesized silver nanocomposites, BioAgNCs) and separated fractions in water (1^I^, 2^I^, 3^I^) and sodium azide (1, 2, 3) systems of one-, two-, and multi-silver ions with peptides and amino acids

*MS* mass spectrometry, *PBS* phosphate-buffered saline, *Abu* 2-aminobutyric acids, *AECys* aminoethyl cysteine, *AP* alanine (amino acids top-down sequencing (AATDS)), *CcPP* CisCarbProPro (AATDS), *Cmc* Carboxy methyl cysteine, *CP* Cysteine (AATDS), *Cys* (*O3H*) Cysteic acid, *Gla* 4- carboxy glutamic acids, *GP* Glycine (AATDS), *HPP* Hystidine (AATDS), *Hyl* Hydroxy lysine, *I/LP* Iso-/Leucine (AATDS), *I/LPP* Iso-/Leucine (AATDS), *iA* Alanina (Amino Acids- i-Types (AAiT)), *iC* Cysteine (AAiT), *iD* Aspartic Acid (AAiT), *iH* Histidine (AAiT), *iK* Lysine (AAiT), *iL* Leucine (AAiT), *iM* Methionine (AAiT), *iP* Proline (AAiT), *iS* Serine (AAiT), *iT* Threonine (AAiT), *MP* Methionine (AATDS), *NP* Asparagine (AATDS), *VPP* Valine (AATDS), *YPP* Tyrosine (AATDS) (According FlexControl, Bruker Daltonics, description)

## Discussion

AgNPs have been widely investigated for their toxicity and cytotoxicity. The most common topic of new research focuses on size-dependent toxic effects and the role of the organic coating. Saenmuangchin et al. [26] reported a separation method for coated AgNPs that involved FFF. They created AgNPs of different sizes coated with citrate and polyvinylpyrrolidone (PVP) and then separated them by size with use of different techniques. This encouraged us to perform more experiments that will improve our understanding of the behavior of silver composites, especially those obtained in biological synthesis, and the role of the composition of the liquid in which they are suspended. For this reason, taking into consideration that the silver biocolloids used in this study contain on the surface an organic deposit [12], we performed A4F for their characterization and to study the influence of the organic core on size separation.

DLS/MALLS measurements are performed on similarly sized particle populations, and can therefore provide more reliable particle size characterization. In DLS the fluctuation of the intensity of light scattered by a colloidal dispersion yields information about the hydrodynamic radius of the sample, whereas MALLS acquires the intensity of light scattering at several different angles and measures the radius of gyration on the basis of the average molar mass [22]. The hydrodynamic diameter and the radius of gyration of the silver composites were slightly larger in aqueous solution than in the sodium azide system. A large diameter of biocolloids indicates some particle aggregation. In the light scattering method, the presence of small aggregates skews the effective diameter distribution toward smaller diameters. Moreover, different solvents (buffers) change the solvation processes because of the different ionic strengths of the media, which influences also the retention time of the system [11]. In the case of an increase of electrolyte ion concentration, the thickness of the electrical double layer is decreased as a result of nonspecific adsorption of ions, which determines the reduction in the value of this potential. Therefore different sizes of silver composites in various buffer systems were recorded.

Furthermore, the differences between the signals in panels A and B in Fig. 2 are consequences of the high intensity of the first signal, which was automatically generated by the software. This phenomenon is probably connected to the buffer composition and variation of ionic strength. However, the intensity of the second and third fractions is correlated in both cases.

To complement the DLS/MALLS information, TEM measurements were made. Regarding the particle size distribution (Fig. 2), the AgNPs show different dispersion depending on the solvent used. In the case of water, TEM revealed similar agglomeration for the first and second fractions, but for the third fraction good dispersion of AgNPs was observed, with size of 4-45 nm. In contrast, the TEM images do not show such agglomerates in sodium azide. Moreover, the TEM results revealed the presence of silver clusters and even several morphologies, including multitwinned (i.e., decahedra, icosahedra, truncated pyramids, and elongated) [23]. Additionally, the MALDI study also showed that the particles have an uncommon cluster structure that can be described as being composed of two or more silver atoms. Indeed, the organic surface of the nanoparticles can modify their dispersion and agglomeration probability.

Many of the results prove the hypothesis that the toxicity of nanoparticles increases with decreased particle size. Ivask et al. [24] investigated several microbial species and concluded that small nanoparticles (10 nm) had more efficient cell–particle contact than larger ones, which led to higher intracellular bioavailability of AgNPs. Moreover, Gliga et al. [2] established the influence of silver stabilized with PVP and citrate on BEAS-2B cells. They showed that cytotoxicity toward human lung cells is independent of the surface coating only for 10-nm particles. In both studies, the toxic effect of NPs was size and dose dependent. The dose-dependent effect was also observed in our previous study [25] investigating AgNPs synthesized by *Actinomycetes*.

Although biocoated and stabilized AgNPs have almost the same effects against various microbial strains, we cannot exclude that the separation mechanism of stabilized and biocoated AgNPs is different. On the basis of the nature of the carrier solvent, the silver particles are fractionated according to their molecular masses, which are consequently influenced by organic deposits. Nonetheless, this clearly indicates that different types of surface coating exert different influences on the retention behavior of biologically synthesized silver composites. The same phenomenon was observed by Saenmuangchin et al. [26].

The spectroscopy study proved the presence of different active functional groups of organic species on silver biocolloids for all fractions. The displacement of the signal and the occurrence of new signals in all of the collected fractions is connected with the vibrational frequencies of the various chemical structures of the molecules. Moreover, the protonation and deprotonation states often induce significant structural changes [17]. Alternatively, the comparative vibration obtained in both cases (water and 0.09% sodium azide) is related to the affinity of silver biocolloids for the solvents. A vibration may experience a shift in frequency because of a conformational change that alters the electron density of the vibrating bonds or the coupling with other vibrations [17]. Moreover, flexible structures will give broader bands than rigid structures, and the bandwidth is a measure of conformational freedom. For a molecule that is bound to proteins, conformational freedom is a natural consequence of that binding. Binding of molecules may also confer enhanced rigidity on proteins, resulting in changes in the bandwidth [17]. The relevance of these results became much more significant when MALDI-TOF MS was used to prove this phenomenon. One-dimensional and two-dimensional MALDI MS have been used for protein identification, sequencing, and searching for posttranslational modifications such as phosphorylation in casein components [13]. This strategy and knowledge of the isotopic pattern of *d*-electron metals allows the identification of organics binding to ions and clusters of metals such as metal proteins, metallopeptidases, and also metal and metal oxide nanoparticles [27]. Many researchers reported the identification of zinc ions bound to organics and chemically synthesized titanium oxide nanoclusters [28, 29]. In our study, we registered the molecular fingerprint of silver biocolloids consisting of two-silver ([Ag_2_–]^+^) and multi-silver ([Ag_*n*_–]^+^) combinations with organic parts such as proline–asparagine–alanine–serine peptide sequences (Fig. 5) or aspartic acid binding to a Ag_17_ cluster (Table 1). In comparison with our previous study [12], besides the presence of active functional groups and silver clusters, in this research we performed deep characterization and identification of surface organic deposits connected with metals and proved the influence of ionic strength on separation of biologically synthesized silver as a native sample and a separated sample.

The presence of these signals suggests an in-source decay mechanism [30] of biologically synthesized AgNPs. Moreover, the presence of peptides combined with silver suggests biocoating of the metallic core by organics secreted into the medium as polypeptides, proteins, or metabolites such as reductase or bacteriocins produced by lactic acid bacteria [31].

## Conclusions

The use of stabilized AgNPs is of huge interest in medical applications because of their size-dependent toxic effects. In this study, A4F coupled with UV, MALLS, and DLS detection and TEM, FTIR spectroscopy, and MALDI MS were applied for fractionation and deep characterization of silver biocolloids. The results proved the presence of differently sized particles in all fractions. They contained one or more clusters of AgNPs, naturally coated with organic deposits derived from native silver biocolloids. In addition, the discrepancies in size measurements appear to be due to the strong influence of organic coating matrix on the hydrodynamic diameter of particles, which is not visible in the transmission electron microscope. Differences in the agglomeration state and therefore in the silver size distribution depend on the nature of the carrier solvent. This study proves that the separation of silver biocolloids relied not only on the particle size but also on the type and branch size of the surface organic deposit. One-dimensional MALDI MS makes it possible to record the molecular fingerprint spectrum of an analyzed sample, whereas two-dimensional MS spectra resolve the structure of the parent ion by a fragmentation approach.

Therefore, biologically synthesized silver composites could be used in medical applications as an alternative to standard AgNPs that are chemically synthesized and coated/stabilized with an organic matrix (e.g., citrate, PVP) because of their natural origin and lack of toxicity.

## Electronic supplementary material

ESM 1 (PDF 306 kb)
