# Supplementary material for: Physicochemical study of natural fractionated biocolloid by asymmetric flow field-flow fractionation in tandem with various complementary techniques using biologically synthesized silver nanocomposites
Source: Anal Bioanal Chem. 2018 Apr 3;410(11):2837–47. doi: 10.1007/s00216-018-0967-0 (PMC5887009; doi:10.1007/s00216-018-0967-0)
Supplement: Supplementary file 1 — (PDF 306 kb) [file 216_2018_967_MOESM1_ESM.pdf]

Analytical and Bioanalytical Chemistry

Electronic Supplementary Material

Physicochemical study of natural fractionated biocolloid by asymmetric flow field-flow fractionation in tandem with various complementary techniques using biologically synthesized silver nanocomposites

Viorica Railean-Plugaru, Pawel Pomastowski, Tomasz Kowalkowski, Myroslav Sprynskyy, Boguslaw Buszewski

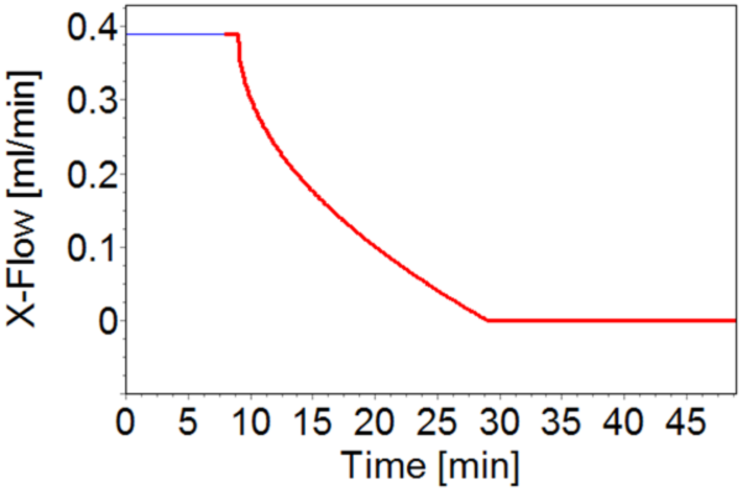

Fig. S1 Gradient of cross flow during the A4F analyses
